# Microbiota Assessment of Fresh-Cut Apples Packaged in Two Different Films

**DOI:** 10.3390/microorganisms11051157

**Published:** 2023-04-28

**Authors:** Joana Madureira, Sara Gonçalves, Celestino Santos-Buelga, Fernanda M. A. Margaça, Isabel C. F. R. Ferreira, Lillian Barros, Sandra Cabo Verde

**Affiliations:** 1 Centro de Ciências e Tecnologias Nucleares (C2TN), Instituto Superior Técnico, Universidade de Lisboa, EstradaNacional 10 ao km 139.7, 2695-066 Loures, Portugal; joanamadureira@ctn.tecnico.ulisboa.pt (J.M.); fmargaca@ctn.tecnico.ulisboa.pt (F.M.A.M.); 2 Centro de Investigação de Montanha (CIMO), Instituto Politécnico de Bragança, Campus de Santa Apolónia, 5300-253 Bragança, Portugal; iferreira@ipb.pt (I.C.F.R.F.); lillian@ipb.pt (L.B.); 3 Laboratório Associado para a Sustentabilidade e Tecnologia em Regiões de Montanha (SusTEC), Instituto Politécnico de Bragança, Campus de Santa Apolónia, 5300-253 Bragança, Portugal; 4 Grupo de Investigación en Polifenoles (GIP-USAL), Facultad de Farmacia, Universidad de Salamanca, Campus Miguel de Unamuno s/n, 37007 Salamanca, Spain; csb@usal.es; 5 ESTeSL-Escola Superior de Tecnologia da Saúde de Lisboa, Instituto Politécnico de Lisboa, 1990-096 Lisboa, Portugal; 6 Unidad de Excelencia Producción, Agrícola y Medioambiente (AGRIENVIRONMENT), Parque Científico, Universidad de Salamanca, 37185 Salamanca, Spain; 7 Departamento de Engenharia e Ciências Nucleares, Instituto Superior Técnico, Universidade de Lisboa, Estrada Nacional 10, ao km 139.7, 2695-066 Loures, Portugal

**Keywords:** minimally processed apple, natural extracts, packaging, natural microbiota

## Abstract

The aim of this work was to assess the natural microbiota of packed fresh-cut apples during refrigerated storage. Two different films were tested for the package, a biodegradable (PLA) film and a conventional and commercial one (OPP). Two antioxidant additives were applied, a natural olive pomace extract and the commercial ascorbic acid used by the industries. The results revealed lower bacteria counts in samples with olive pomace extract and PLA films than in those with ascorbic acid and OPP films after 5 and 12 days of storage. These findings suggest that the use of such natural extracts as additives in fruits could delay the growth of mesophilic bacteria. The characterization and identification of the bacterial isolates from fresh-cut apple samples showed that the most prevalent species were *Citrobacter freundii*, *Staphylococcus warneri*, *Pseudomonas oryzihabitans*, *Alcalinogenes faecalis*, *Corynebacterium jeikeium*, *Micrococcus* spp., *Pantoea aglomerans* and *Bacillus* spp. Furthermore, an increase in the microbial diversity during the storage time at refrigerated temperatures was observed, except for the sample treated with olive pomace extract and packaged in OPP film. The highest microbial diversity was found for samples with ascorbic acid as an additive. This could indicate a negative effect of ascorbic acid on the microbial inhibition of apple slices. The natural olive pomace extract demonstrated potential as an antimicrobial additive for fresh-cut apples.

## 1. Introduction

Fruits are excellent sources of nutrients, being an essential part of a healthy diet due to their nutritional qualities. The demand of consumers for minimally processed foods that preserve their safety and high quality attributes has been growing. This motivates food industries to look for innovative processes by which to manufacture these kinds of products. Ready-to-eat products, such as fresh-cut apples, are very perishable and susceptible to spoilage. This is because they can be fast oxidized and are susceptible to contamination during processing and storage [1,2]. Some of the microorganisms involved can cause food spoilage and/or may be potentially hazardous to human health related to different illnesses. Therefore, it is important to understand their incidence and survival during the storage period [3]. Regarding the apple fruits, different apple tissues present bacterial communities that vary in diversity and abundance. On the other hand, the global core microbiome of apple fruit has been considered an important contributor to human gut microbiome, stimulating the human immune system [4].

Synthetic antioxidants, such as butylated hydroxyanisole (BHA), butylated hydroxytoluene (BHT) and ascorbic acid, have been widely used by food production industries in food products. However, several studies have described relationships between their use and some harmful effects on health [5,6,7,8]. Engin et al. [5] reported liver toxicity associated with the use of BHT in endotoxemia settings, whereas other authors have reported the carcinogenic effect of both antioxidants in experimental animals [7,8]. Jeong et al. [6] found that high doses of BHA had endocrine-disruption effects that altered the development and functions of the reproductive systems of male and female rats including sex organ weights and sexual maturation.

In this sense, the search for natural additives to replace the synthetic antioxidants and antimicrobials as food preservatives has attracted increasing interest [9,10,11]. In recent years, special attention has been paid to natural additives that can be extracted from the wastes of agricultural food industries [12]. One candidate is olive pomace, the main waste generated by the olive oil industry. It is considered a rich source of bioactive compounds with putative health benefits for humans [10]. Moreover, previous studies demonstrated that olive pomace extracts provided antioxidant and antimicrobial potential against different microorganisms [13,14]. The high antidiabetic and anti-inflammatory activities of such extracts was recently reported for the first time, with a high cytotoxic effect for the breast adenocarcinoma (MCF-7) cell line [13]. In this way, these extracts could possibly be used by the industries as a new ingredient to produce functional foods with added value. Some researchers have been exploring this application [15,16,17,18]. The addition of dried olive pomace to bread, pasta and granola bars increased their total phenolic compounds [18], improving the nutritional quality of bread without compromising the product acceptability [19]. *Taralli*, a typical Italian bakery product, enriched with 20% fermented olive pomace, presented enhanced bioactive compounds content and maintained the low amounts of saturated fatty acids during storage [17]. On the other hand, Cedola et al. [16] suggested that although the enriched fish burgers with 10% dry olive pomace flour were not very acceptable due to a bitter and spicy taste, they presented high amounts of total phenolic compounds, total flavonoids and, consequently, an increase in antioxidant activity. The incorporation of olive pomace in yogurts also provided additional and essential health benefits, such as the increase in unsaturated fatty acids and the greater bioaccessibility of total phenolics after in vitro digestion [20].

Based on these observations, the aim of this work was to compare the inhibition effect on the microbial population of fresh-cut apples preserved using ascorbic acid—a commercial additive—and an olive pomace extract—a natural preservative—during 12 days of refrigerated storage in two different packaging bags composed of biodegradable polylactic acid (PLA) film and conventional oriented polypropylene (OPP) film. In our previous studies, the optimization of the extraction of olive pomace compounds was investigated. It was found that the application of ionizing radiation at 5 kGy dose to olive pomace improved the extractability of its bioactive compounds 2-fold [14], with enhanced bioactive properties [13]. Thus, the olive pomace extracts used to treat fresh-cut apples were obtained following the optimized procedure. A comprehensive characterization was performed in order to ascertain the most frequent bacteria in the samples of fresh-cut apples.

To the best of our knowledge, this study represents the first assessment of the use of olive pomace extract as a natural preservative on fresh-cut apples. We believe this work can contribute to encouraging the food industries to develop new functional foods and to promote the economic and environmental sustainability of the olive oil sector.

## 2. Materials and Methods

### 2.1. Olive Pomace Samples and Irradiation Experiments

Olive pomace samples were collected from UCASUL (União de Cooperativas Agrícolas do Sul, Alvito, Portugal). The samples were submitted to gamma radiation treatment in triplicate, using an absorbed dose of 5 kGy [14].

### 2.2. Olive Pomace Natural Ingredients: Phenolic Extracts Preparation

The extraction of phenolic compounds from irradiated olive pomace was performed using the optimal conditions of heat-assisted extraction obtained by Madureira et al. [21]. The selected extracts were those with the higher bioactive properties [13]. Sample extraction was performed in triplicate.

### 2.3. Preparation of Minimally Processed Apples

‘Royal Gala’ apples were purchased from a local supermarket in Lisbon (Portugal). First, they were washed, dried and cut into eight equal slices. Then, two groups of samples were prepared by immersion during 1 min in: olive pomace extract solution (EXT, 0.315%, *w*/*v*) for one group, and in ascorbic acid solution (AA, 0.315%, *w*/*v*) for the other. After that, three slices of each group were packed into biodegradable polylactic acid (PLA) (Vegware, Edinburgh, UK) and oriented polypropylene (OPP) (Campotec S.A, Torres Vedras, Portugal) film bags (10 × 10 cm) and sealed. The packs were then stored during 12 days in controlled chambers at 4 °C and 85% relative humidity. PLA biodegradable films were produced from corn starch, while the OPP films were conventional and commercial film plastic bags. Three independent packages were analyzed for each group on each day.

### 2.4. Characterization of Natural Mesophilic Bacteria in Fresh-Cut Apples

The fruit slices were analyzed over storage time (at 0, 5, 12 days of storage at 4 °C) for their microbial load and further phenotypic characterization. Briefly, apple slices from each package (~150 g) were homogenized in a stomacher (Stomacher 3500; Seaward, Peterlee, UK) for 15 min using 100 mL of buffered peptone water. Afterwards, serial decimal dilutions were prepared and inoculated in triplicate in Tryptic Soy Agar (TSA) plates. The plates were incubated at 30 °C for 7 days. Colony Forming Units (CFU) were counted, and the results were expressed as Log colony-forming units per gram of fresh fruit (Log CFU/g).

Bacterial isolates were phenotyped based on Bergey’s Manual of Determinative Bacteriology [22]. The bacterial isolates were identified using RapID Systems (Remel, Thermo Scientific).

### 2.5. Statistical Analysis

Results were expressed as mean ± standard deviation. The differences between additives and packaging were analyzed using one-way analysis of variance (ANOVA) followed by Tukey’s HSD test with α = 0.05. A significance level of *p* < 0.05 was considered. The bacterial diversity indices were estimated for bacterial species detected in fresh-cut apple samples by storage time and packaging film.

The Simpson’s index of diversity (1-D) was calculated based on the following Equation (1):

(1) $$1-D=1-\left(\frac{\sum n\left(n-1\right)}{N\left(N-1\right)}\right),$$

where *n* is the total number of isolates of a certain species and *N* is the total number of isolates of all species [23].

The Shannon diversity index (*H*) was calculated by the Equation (2):

(2) $$H=-\sum \left(p_{i}\times lnp_{i}\right),$$

where *p_i_* is the proportion of the entire microbial community made up of species *i* [24]

## 3. Results and Discussion

The bacterial load present in fresh-cut apple samples was assessed immediately after packaging the samples (T0) and after 5 days (T5) and 12 days (T12) of storage at refrigerated temperatures (4 °C). This was conducted in order to establish both the efficiency of the treatment with each antioxidant solution and the effect of packaging in different films.

**Figure 1:**
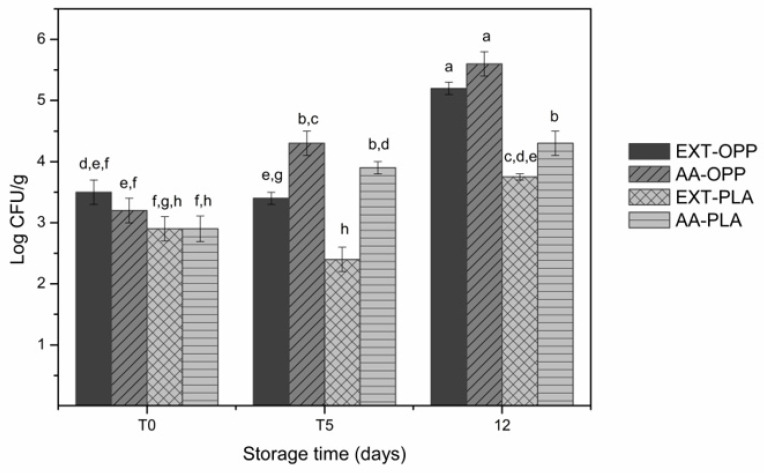
Aerobic mesophilic bacterial counts for fresh-cut apple samples (EXT-OPP: treated with olive pomace extracts and packaged in OPP films; AA-OPP: treated with ascorbic acid and packaged in OPP films; EXT-PLA: treated with olive pomace extracts and packaged in PLA films; AA-PLA: treated with ascorbic acid and packaged in PLA films) immediately after treatment and packaging (T0) and after 5 (T5) and 12 (T12) days of refrigerated storage. Means with equal lowercase letters are not statistically different by Tukey’s test with 5% significance level.

As expected, immediately after immersion into the antioxidant solutions and packaging (T0), no significant differences (*p* > 0.05) were observed in the bacterial concentrations of all the analyzed samples (Figure 1). The fresh-cut apples presented an aerobic bacterial mesophilic population ranging from 2.9 ± 0.2 log CFU/g to 3.5 ± 0.2 log CFU/g. This is coherent with the average levels of aerobic mesophilic bacteria ranging from 2 to 4 log CFU/g reported by other authors for fresh-cut apple samples [25,26]. On the other hand, the results found in the present work are lower than those described by Graça et al. [2] for fresh-cut apple samples that varied from 3.3 to 8.9 log CFU/g.

During refrigerated storage, different trends were observed depending on the antioxidant treatment and packaging film. For samples treated with ascorbic acid solution, packed in OPP film, the mesophilic bacterial population of fresh-cut apples significantly increased (*p* < 0.05) over time. In this case (AA-OPP), the results were: 4.3 ± 0.2 log CFU/g and 5.6 ± 0.2 log CFU/g for T5 and T12, respectively. While for those packed in PLA film (AA-PLA), the results after 5 days of storage (T5) were 3.9 ± 0.1 log CFU/g, remaining roughly constant until the end of the experiment (T12): 4.3 ± 0.2 log CFU/g. For samples treated with olive pomace extract, both OPP and PLA films were able to maintain the bacterial concentrations during 5 days of refrigerated storage (3.4 ± 0.1 log CFU/g and 2.4 ± 0.2 log CFU/g, respectively). However, a significant increase was observed after 12 days of storage at 4 °C (5.2 ± 0.1 log CFU/g and 3.75 ± 0.05 log CFU/g, for OPP and PLA films, respectively).

The samples with the largest counts (Figure 1) are those with ascorbic acid as the additive and OPP film as the packaging bag (AA-OPP). These samples, with 5.6 ± 0.2 log CFU/g, are the ones closer to the Portuguese recommended limit for mesophilic bacteria in ready-to-eat foods that is 6 log CFU/g [27]. The overall results demonstrated that the lower bacteria counts were achieved for the samples using olive pomace extracts packed in PLA films. Those showed 2.4 ± 0.2 log CFU/g after 5 days and 3.75 ± 0.05 log CFU/g after 12 days of refrigerated storage. This finding highlights the combined use of the natural pomace extracts and PLA films packaging to efficiently delay the growth of mesophilic bacteria in fruits.

**Figure 2:**
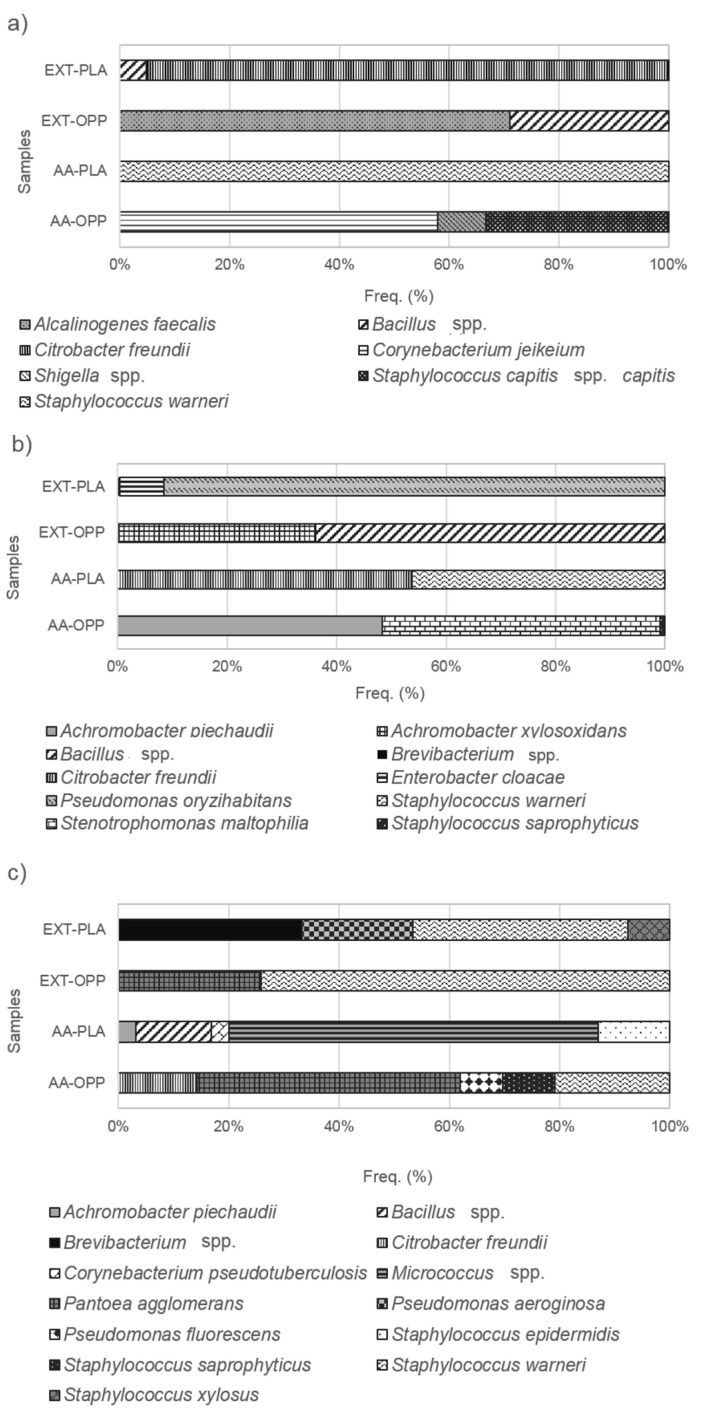
Relative abundances (%) of bacterial species isolated from the fresh-cut apple samples (EXT-OPP: treated with olive pomace extracts and packaged in OPP films; AA-OPP: treated with ascorbic acid and packaged in OPP films; EXT-PLA: treated with olive pomace extracts and packaged in PLA films; AA-PLA: treated with ascorbic acid and packaged in PLA films): (**a**) immediately after treatment and packaging (T0), (**b**) after 5 (T5) days of refrigerated storage and (**c**) after 12 (T12) days of refrigerated storage.

The quantification and identification of the bacterial isolates from fresh-cut apple samples were performed using culture-based methods (Figure 2).

Immediately after packaging (T0), the most prevalent bacterial species identified in the analyzed samples were *Citrobacter freundii* (95%) in EXT-PLA, *Alcalinogenes faecalis* (71%) in EXT-OPP, *Staphylococcus warneri* (100%) in AA-PLA and *Corynebacterium jeikeium* (58%) in AA-OPP. The first two species are mostly found in the environment, while the other two are part of the skin microbiota, and all of them could be recognized as opportunistic pathogens. *C. freundii* is a Gram-negative bacterium of the *Enterobacteriaceae* family. Although it is considered non-pathogenic when interacting with healthy individuals, it can cause a life-threatening infection that may progress into sepsis when colonizing the bloodstream [28]. *A. faecalis* is a potentially emerging pathogen for hospitalized patients and it usually causes opportunistic infections in humans such as meningitis [29], peritonitis [30] or pneumonia [31]. Moreover, its resistance to several antibiotics has been increasing, turning difficult the treatment of its infections [32,33]. *C. jeikeium* is a highly virulent pathogen and resistant to antimicrobial agents. One-third of immunocompromised patients with *C. jeikeium* infection present pulmonary lesions [34]. Additionally, the infections by this microorganism can also promote rash and skin lesions [35]. *S. warneri* is a commensal microorganism on skin flora, resistant to penicillins that rarely causes infection in healthy individuals. However, two cases were reported of urinary tract infections in a patient with liver cirrhosis [36], as well multiple subcutaneous abscesses in an immunocompetent individual [37] caused by *S. warneri* infections. This microorganism had been previously isolated from fresh apples and identified by the amplification of the 16S rRNA gene [38].

After 5 days (T5) of packaging and refrigerated storage, the most prevalent bacterial species identified in the samples were *Pseudomonas oryzihabitans* (92%) in EXT-PLA, *Bacillus* spp. (64%) in EXT-OPP, *C. freundii* (54%) and *S. warneri* (46%) in AA-PLA and *Stenotrophomonas maltophilia* (51%) and *Achromobacter piechaudii* (48%) in AA-OPP (Figure 2). The majority of *Pseudomonas* species are recognized as psychrotolerant or psychrotrophic (growing below 15 °C), justifying their prevalence on refrigerated products [39]. The abundance and persistence of *Pseudomonas* in foods could also be related to its ubiquity in the environment, and, most importantly, to its ability to form biofilm, which may increase its tolerance to adverse conditions, including several antimicrobial treatments [40]. *P. oryzihabitans* is frequently found in hospital environments. It is rarely implicated in human infections. However, it was reported as being the cause of an infection in a 72-year-old man who was apparently healthy after a total hip arthroplasty four years before [41], as well as multiple skin rashes on the scalp and neck of a 1-year-old girl from Ghana [42]. Nevertheless, *P. oryzihabitans* is easy to eradicate due to its susceptibility to antibiotic therapy. Several *Bacillus* species have been associated with a variety of infections as some of them are considered foodborne pathogens, such as *B. cereus*, *B. subtilis*, *B. anthracis* and *B. licheniformis* [43]. At the same time, *Bacillus* spp. (*B. cereus*, *B. licheniformis*, *B. subtilis*, *B. clausii*, *B. coagulans*, *B. polyfermenticus* and *B. pumilus*) are being used as probiotics due to their ability to form endospores, to replace antibiotics and to act as immune-modulators and microbiome regulators [44]. *S. maltophilia* is an environmental multiple-drug-resistant organism that has been associated with nosocomial and community-acquired infections (e.g., pneumonia, meningitis, bacteremia and endocarditis) described by Brooke [45]. *A. piechaudii* is a recently described Gram-negative bacteria usually found in soil and water with low pathogenicity. Nevertheless, a few cases of *A. piechaudii* infections have been reported, namely, bacteremia, both in a neutropenic man with hematologic malignancy [46] and in an immunocompetent host [47].

After 12 days (T12) of packaging and refrigerated storage, the most prevalent bacterial species identified in the samples were *S. warneri* in EXT-PLA (39%) and EXT-OPP (74%), *Micrococcus* spp. (67%) in AA-PLA and *Pantoea agglomerans* (48%) in AA-OPP (Figure 2). *Micrococcus* species, members of the family *Micrococcaceae*, are usually regarded as contaminants from skin and mucous membranes, but they are not considered to be pathogenic. Nonetheless, some authors related them with some infections in immunocompromised patients, namely, pneumonia in a 26-year-old female patient with acute myeloid leukemia [48], catheter-related infection in hypertension patients [49], brain abscess in a patient with systemic lupus erythematosus [50] and native valve endocarditis in a non-Hodgkin’s lymphoma patient [51], all caused by *M. luteus* infection. Infections by *P. agglomerans*, an environmental and agricultural organism of the *Enterobacteriaceae* family, are uncommon and an adequate treatment with antibiotic promotes a full recovery of the patients [52]. In spite of that, this bacteria is an opportunistic pathogen associated with wound- or hospital-acquired infections, occurring mostly in immunocompromised individuals [53,54,55]. *P. agglomerans* was only found at T12 and for the samples packaged in OPP films. This could indicate the lower ability of this film to control the bacteria growth owing to its permeability characteristics. Previously, Torres et al. [56] reported high viability of *P. agglomerans* in packages with low oxygen permeability. However, this is not in agreement with the results of this work since OPP films presented higher oxygen permeability (2.68 × 10^−12^ m^2^/s) when compared to PLA films (2.97 × 10^−16^ m^2^/s).

According to the literature, the bacteria frequently associated with the spoilage of minimally-processed fruit and vegetables belong to the genera *Corynebacterium*, P*seudomonas*, *Erwinia* and other *Enterobacteriaceae* [40]. Bacterial isolates from these genera and *Enterobacteriaceae* family were isolated in the apple samples. Nevertheless, these were not the most prevalent bacteria at the end of storage period. In fact, after 12 days of refrigerated storage, the fresh-cut apples in PLA film presented *Micrococcus* spp. (AA-PLA, 67%) and *S. warneri* (EXT-PLA, 39%) as the most frequent isolate bacteria (Figure 2c).

The bacterial community in a specific environment could be characterized by the number of species present and their numerical composition, the bacterial diversity. In general, an increase in the number of detected species of aerobic mesophilic bacteria was observed in apple samples stored for 12 days (Figure 2). This can be attributed to the cutting of the apples before immersion in antioxidant solutions and packaging. This procedure increases the water activity and availability of nutrients at cut surface [57], causing tissue damages and promoting the growth of microorganisms [3]. Nonetheless, the sample with olive pomace extract as additive and packaged with OPP film (EXT-OPP) maintained the number of bacterial species during the storage period in contrast with the other samples. By contrast, the highest number of bacterial species was found in the samples using ascorbic acid as additive packaged in either film (AA-PLA and AA-OPP). This suggests that the commercial additive ascorbic acid could have a limited effect to control the microbial diversity in fruits.

A variety of indices have been developed for the comparison of the bacterial diversity [58]. Among them, the most commonly used in bacterial diversity measurements are Shannon–Weaver and Simpson diversity indices [23,24].

Simpson’s index of diversity (1-D) indicates the number of different species isolated and how evenly these different species are distributed based on total detected microorganisms. The Shannon index is a quantitative indicator of the number of different bacterial species that are present in a sample, considering the uniformity in the distribution of these bacteria in these species. The Shannon diversity index takes a greater weight on species richness (number of different species present). In turn the Simpson index contemplates species evenness (the relative abundance of the different species consisting of a community) more than species richness in its estimative [58]. The calculated Simpson diversity and Shannon indices for the bacterial community detected in the fresh-cut apple samples during storage with the two different packaging films and additives are presented in Table 1.

Shannon’s and Simpson’s indices showed similar trends, the higher the indices’ values, the higher the bacterial community diversity. The diversity indices analysis indicated an increasing trend in the bacterial community diversity of fresh-cut apples with storage time (Table 1), as mentioned above (Figure 2).

The bacterial diversity measured by the Simpson and Shannon indices showed differences among the four types of processed (package film-additive) fresh-cut apples. The lowest bacterial diversity was obtained for fresh-cut apples treated with olive pomace extract and packaged in PLA film. The samples with olive pomace extract demonstrated less bacterial diversity comparatively with the ones treated with ascorbic acid. The fresh-cut apple bacterial community composition was indicated to be influenced by the additive and film on the package. This suggests that the combination of the treatment with olive pomace extract and packaging with PLA film could be able to control the bacterial diversity in apple slices.

None of the analyzed samples were positive for *Escherichia coli*, *Cronobacter sakazakii*, *Salmonella* spp., *Clostridium perfringens* and *Listeria monocytogenes*, the main foodborne pathogens associated with fresh fruit [3]. Previous studies also described the low or inexistent presence of foodborne microorganisms in fresh fruits [2,59]. Adi et al. [60] reported that the majority of the cultivated bacteria in apples belonged to *Bacillus*, *Curtobacterium*, *Erwinia*, *Pseudomonas* and *Xanthomonas* genera. Abdelfattah et al. [4] cited *Sphingomonas* and *Methylobacterium* as the main bacterial genera of apple microbiome. Leishman et al. [61] also reported the survival of *B. anthracis* spores in fruit juices for at least a month.

In the present study, the species *S. warneri* and the genus *Bacillus* were found to be the most common bacteria over all the analyzed periods, T0–T12 (Figure 2). López-González and co-workers [62] described the antagonistic activity of *Bacillus* species isolated from ‘Royal Gala’ apples against *Penicillium expansum*. This might be related to their capacity to produce lipopeptides and siderophoros. *B. mojavensis* EGE-B-5.2i and *B. thuringiensis* EGE-B-14.1i were reported to be efficient antifungal agents against *Aspergillus niger* of Turkish figs [63]. Besides being the most prevalent species found in the analyzed samples (*S. warneri*), *Staphylococcus* genus has many pathogenic species often found in the skin. Previous studies indicated that *Staphylococcus* were abundant on minimally processed fruit surfaces and its detection could indicate human cross-contamination [53]. Herein, other *Staphylococcus* species were found at frequencies lower than 33%, such as S. *capitis* ssp. *capitis*, *S. saprophyticus*, *S. xylosus* and *S. epidermidis*. Some of these species are associated with the skin microbiota and, consequently, to the human microbiome, being essential to maintaining human health and preventing diseases. For instance, *S. epidermidis* was reported as the producer of 6-N-hydroxylaminopurine (6-HAP), which can protect against neoplasia [64] and act as a stimulator of nasal epithelia to antimicrobial peptides, killing competing pathogens [65]. Recently, Adi et al. [60] suggested that some specific bacteria present in raw-eaten fruits may have beneficial properties for human health and that some of the bacteria associated with apples can be compatible with the gastrointestinal environment.

The obtained results indicated a diverse microbial pattern for fresh-cut apples depending on the type and length of storage as influenced by a series of factors, including packaging and processing conditions.

## 4. Conclusions

In recent years, the growing interest in a healthy and convenient diet has led to a significant increase in the consumption of prepacked ‘ready-to-eat’ fruits. In this work, an assessment of the natural microbiota of apple slices treated with two different antioxidants and packed in bags made of two different films was performed during 12 days of refrigerated storage. The antioxidants were ascorbic acid and olive pomace extract, and the bags were made of a biodegradable (PLA) film and a conventional one (OPP). The results revealed lower bacteria counts in the samples using olive pomace extracts and PLA films after 5 and 12 days of storage when compared with ascorbic acid and OPP films. These findings suggest that the use of those natural extracts as additives in fruits could be efficient to delay the growth of mesophilic bacteria. The identification of the bacterial isolates from fresh-cut apple samples indicated that none of the apple samples were positive for the main foodborne pathogens associated with fresh fruit. Nevertheless, the most prevalent species of the analyzed samples were *Citrobacter freundii*, *Staphylococcus warneri*, *Pseudomonas oryzihabitans*, *Alcalinogenes faecalis*, *Corynebacterium jeikeium*, *Micrococcus* spp., *Pantoea aglomerans* and *Bacillus* spp. These are bacteria from human and environmental microbiota. It was possible to observe an increase in microbial diversity during the storage period. The highest microbial diversity was found in the samples using ascorbic acid as an additive (AA-PLA and AA-OPP). This suggests that the commercial additive, ascorbic acid, may have a limited effect on the microbial inhibition in the apple slices. As a global conclusion, it can be stated that the olive pomace extract demonstrated the potential to be used as a natural antimicrobial preservative in fresh ready-to-eat food. Furthermore, its combination with PLA film could be the most adequate process, as confirmed by Shannon’s and Simpson’s indices. The obtained results can assist the food industries in their efforts both to generate new functional foods and to promote the economic and environmental sustainability of the olive oil sector.

## Figures and Tables

**Table 1:** Diversity indices of bacteria from fresh-cut apple samples by storage time (T0, T5 and T12) or by packaging film (OPP and PLA) and additive (ascorbic acid and olive pomace extract).

Fresh-Cut Apples ^1^	Simpson’s Index of Diversity (1-D)	Shannon Diversity Index (H)
T0	0.60	1.29
T5	0.71	1.56
T12	0.79	1.87
AA-PPO	0.84	1.98
AA-PLA	0.70	1.51
EXT-PPO	0.60	1.18
EXT-PLA	0.47	1.02

^1^ T0: microbial community immediately after treatment and packaging; T5: microbial community after 5 days of refrigerated storage; T12: microbial community after 12 days of refrigerated storage. AA-OPP: treated with ascorbic acid and packaged in OPP films; AA-PLA: treated with ascorbic acid and packaged in PLA films; EXT-OPP: treated with olive pomace extracts and packaged in OPP films; EXT-PLA: treated with olive pomace extracts and packaged in PLA films.

## Data Availability

The datasets used/or analyzed during the current study are available from the corresponding author on reasonable request.
